# Editorial: New insights in *Chlamydia*: host interactions and pathogenesis

**DOI:** 10.3389/fcimb.2023.1251582

**Published:** 2023-08-17

**Authors:** Hector Alex Saka, Maria Teresa Damiani

**Affiliations:** ^1^ National Council on Scientific and Technical Research (CONICET) Centre for Research in Clinical Biochemistry and Immunology (CIBICI), Cordoba, Argentina; ^2^ Department of Clinical Biochemistry, School of Chemical Sciences, National University of Cordoba, Cordoba, Argentina; ^3^ National Council on Scientific and Technical Research (CONICET) Institute of Medicine and Experimental Biology of Cuyo (IMBECU), Mendoza, Argentina; ^4^ Faculty of Medical Sciences, Institute of Biochemistry and Biotechnology, National University of Cuyo (UNCUYO), Mendoza, Argentina

**Keywords:** *Chlamydia*, intracellular bacteria, pathogen-host cell interaction, bacterial virulence, sexually transmitted infections

*Chlamydia* are ancient and successful pathogens, evolutionary distinct from most bacteria and highly adapted to an intracellular lifestyle. To propagate, *Chlamydia* deploy a plethora of resources oriented to fulfill different steps of their pathogenic strategy, including infection and invasion of their host cells, the transition from infectious elementary bodies to replicative reticulate bodies, establishing an intracellular replicative niche inside a membrane-enclosed compartment (the “inclusion”), transition back to elementary bodies and finally, exit of the host cell. Each of these steps involves complex and poorly understood interactions with their hosts.

As obligate intracellular pathogens, *Chlamydia* have evolved many strategies to acquire nutrients and manipulate cell pathways to evade the anti-bacterial responses elicited by the host. Technical advances in imaging, high-throughput “omics”, sequencing, and genetic tools have contributed to unraveling some of the chlamydial adaptive mechanisms and virulence factors. The ability to manipulate the chlamydial genome, which has been an unreachable challenge until only a few years ago, opened the door to elucidate, at the molecular level, the chlamydial factors relevant for the intracellular lifestyle, the pathogenic mechanisms, and immune evasion that these bacteria use to establish acute or persistent infections. Discovering how *Chlamydia* exploit host cell signaling, vesicular transport, and metabolism will help to unravel obligate intracellular bacterial survival mechanisms. Gathering recent breakthroughs in *Chlamydia* biology, pathogenesis, interactions with host cells, strategies for intracellular survival, persistence, and bacterial mechanism for immune response evasion constitutes a key platform for developing novel preventive and therapeutic anti-chlamydial approaches, including vaccines. A detailed assessment of bacterial proteins, effectors and enzymes involved in any step of *Chlamydia* development (cell invasion, inclusion development, replication and exit from host cells) is crucial for understanding the rampant success of *Chlamydia* intracellular lifestyle. Moreover, the characterization of *Chlamydia* persistence is critical for expanding current information about chronic infections and relapse after treatment. In this context, uncovering the molecular basis of *Chlamydia*-host interactions opens the door to identifying novel targets to control chlamydial infections. Furthermore, studies to increase available data about chlamydial strategies to subvert adaptive and innate immune responses certainly will contribute to designing preventive vaccines.

In this Research Topic, seven papers address different aspects and new developments in the *Chlamydia*-Host interactions and pathogenesis field.

Triboulet et al. demonstrated that the *Chlamydia trachomatiss* protein CT295 is a phosphoglucomutase that mediates the conversion of glucose-1-phosphate into glucose-6-phosphate in the lumen of the chlamydial inclusion. This reaction plays a crucial role in the catabolism of glycogen molecules that accumulate in the inclusion lumen of *C. trachomatis*, *C. muridarum*, and *C. suis*. Interestingly, the authors identified a type three secretion (T3S) signal sequence in CT295 recognized by the T3S machinery of *Shigella*. Thus, to catabolize accumulated glycogen, CT295 chlamydial phosphoglucomutase may access the inclusion lumen *via* the chlamydial T3S machinery.

Pereira et al. studied the role of CteG, a previously described T3S effector of *C. trachomatis* shown to localize in the plasma membrane at late stages post-infection. Taking advantage of a CteG-null mutant, these authors observed that lack of CteG resulted in a defective chlamydial lytic exit from the host cell. A plasmid expressing CteG can rescue the normal phenotype. Thus, this study identified the first chlamydial T3S effector involved in lytic exit from the host cell.

Wang et al. contributed a review article addressing the role of tryptophan in *C. trachomatis* persistence. In this review, the authors compiled relevant findings related to critical aspects of persistent *Chlamydia*, including the biosynthesis and regulation of tryptophan in the host cell, the influence of tryptophan on *C. trachomatis* infection, and the relationship of *C. trachomatis* persistence with the tryptophan/IFNɣ axis.

Gravitte et al. investigated the impact of the hormonal environment in the *Chlamydia muridarum* infection *in vivo*. These investigators used ovariectomized mice to study how the administration of estrogen influences the progression of *C. muridarum* in vaginal infections and the immune response against this pathogen. Also, these authors used mice knock-outs for estrogen receptors ERα and ERβ to investigate the impact of these receptors on *C. muridarum* infection and immune response. The main findings of this study indicated that the hormonal environment altered T cell recruitment and IFNƐ production in the genital tract of both infected ovariectomized and sham mice. Additionally, the lack of ERα impaired the shedding of *C. muridarum* and resulted in faster clearance of the infection, which correlated with an increased number of regulatory T-cells and higher expression of IFNƐ at early times post-infection in these mice.

Wang and Wang contributed a review article focused on the role of leukemia inhibitory factor (LIF) in chlamydial pathogenesis. LIF is a member of the IL-6 cytokine family that is induced by *C. trachomatis* infection. This review addresses the biology and physiological role of LIF and its receptor (LIFR) and analyzes the potential effects of LIF/LIFR-mediated signaling in *C. trachomatis* pathogenesis. The authors found that the available evidence indicates that the production of LIF aims to maintain epithelial homeostasis and tissue repair in the context of *C. trachomatis* infection. However, the prolonged LIF/LIFR-mediated signaling results in a harmful transformation of the microenvironment of the fallopian tube and the cellular composition of the epithelium, which may lead to an increased risk of infertility, ectopic pregnancy, and cancer.

Caven et al. performed a transcriptomic analysis of *Chlamydia*-infected epithelial cells and found that target genes of the host protein YAP, a transcriptional coactivator involved in cell proliferation, wound healing, and fibrosis, are induced during chlamydial infection. These findings correlated with increased nuclear translocation of YAP and *de novo* protein synthesis during mid-cycle infection. The evidence presented in this manuscript supports a *Chlamydia*-directed process resulting in alterations of the transcriptome of infected cells potentially linked to chlamydial fibrosis and sequelae.

Jiang et al. researched if pyroptosis, previously found to be induced during *Chlamydia* infection, impacts chlamydial growth. These authors found that *C. trachomatis* L2-infected RAW 264.7 mouse macrophages underwent pyroptosis accompanied by caspase-1, caspase-11, and gasdermin D activation. Additionally, the results of this manuscript show that pyroptosis induced by *C. trachomatis* resulted in significant inhibition of intracellular chlamydial growth and that the inactivation of caspase-1, caspase-11, or gasdermin D rescued bacterial yields. These findings support the idea that pyroptosis may act as an intrinsic mechanism of the host to restrict *C. trachomatis* intracellular growth.

The understanding of *Chlamydia*-host interactions and pathogenesis is crucial not only for elucidating the microbiology of these intriguing bacteria but also a path for discovering host cell responses and for the design of evidence-based strategies, to mitigate the impact of chlamydial infections on human health. This Research Topic gathers relevant and original discoveries about new bacterial effectors and mechanisms of invasion and host cell exit. In addition, it contains new data about the hijacking of host signaling and metabolism as well as bacterial mechanisms of evasion of host innate and adaptive immune response. Altogether, the findings collected in this Research Topic make valuable contributions aimed toward a better understanding of Chlamydial-host interactions.

## Author contributions

All authors listed have made a substantial, direct, and intellectual contribution to the work and approved it for publication.

